# Virtual Reality Training for Post-Stroke Upper Limb Hemiparesis

**DOI:** 10.3390/neurolint18080149

**Published:** 2026-08-12

**Authors:** Kanta Kitabayashi, Naoki Yoshioka

**Affiliations:** 1 Department of Rehabilitation, International University of Health and Welfare Ichikawa Medical Center, Chiba 272-0827, Japan; kanta@ihwg.jp; 2 Department of Rehabilitation Medicine, Graduate School of Medicine, International University of Health and Welfare, Chiba 286-8686, Japan; 3 Department of Radiology, International University of Health and Welfare Narita Hospital, Chiba 286-8520, Japan

**Keywords:** stroke, rehabilitation, neurorehabilitation, virtual reality, upper limb hemiparesis

## Abstract

In recent years, the application of virtual reality (VR) in rehabilitation medicine has gained increasing attention. VR is generally classified into two types: non-immersive and immersive systems. Non-immersive VR allows patients to interact with virtual environments through screens while maintaining a connection with the real world. Several studies have reported that the combined application of non-immersive VR and adjunctive technologies such as transcranial direct current stimulation and hand exoskeleton devices could facilitate functional recovery of hemiparetic limbs after stroke. In contrast, immersive VR is applied with the use of head-mounted displays to encompass the user’s visual field, providing a high level of immersion and an enhanced sense of presence. This enhanced experience has been shown to promote patients’ attention and motivation, which are important factors in motor learning. Emerging evidence suggests that immersive VR is effective for improving upper limb motor function in post-stroke hemiparetic patients. Furthermore, rehabilitative training performed in immersive virtual environments may facilitate the transfer of acquired motor skills to real activities of daily living. Future research should aim to generate high-quality evidence through large-scale, multicenter randomized controlled trials to better establish the clinical efficacy and optimal implementation of VR-based interventions in stroke rehabilitation.

## 1. Introduction

Virtual reality (VR) is a technology that enables users to experience computer-generated three-dimensional virtual environments as if they were part of the real world. By integrating sensory information, including visual, auditory, and tactile stimuli, VR provides a highly immersive experience. In recent years, the application of VR in healthcare has expanded rapidly, particularly in the field of stroke rehabilitation.

Upper limb motor impairment is one of the most common sequelae of stroke, and many patients continue to experience persistent functional deficits even during the chronic phase [1,2]. Upper limb impairments, including muscle weakness, sensory deficits, impaired motor coordination, impaired fine motor control, and spasticity, not only limit activities of daily living (ADL), such as eating, dressing, and personal grooming, but also reduce the use of the paretic limb, thereby further hindering functional recovery. Consequently, these impairments are associated with reduced quality of life (QOL) for both patients and caregivers, as well as increased socioeconomic burden [3]. Therefore, restoration of upper limb motor function is considered one of the primary goals of stroke rehabilitation.

Although several review articles have summarized the effectiveness of VR-based rehabilitation for post-stroke upper limb hemiparesis, few have specifically focused on the practical characteristics, strengths, limitations, and representative clinical applications of immersive and non-immersive VR. Accordingly, this review provides an overview of the history and classification of VR, followed by a discussion of the theoretical foundations of VR-based rehabilitation, the clinical applications of immersive and non-immersive VR for post-stroke upper limb hemiparesis, and evidence from meta-analyses. Finally, evidence-informed considerations for VR training protocols, practical implementation issues, and future perspectives are discussed.

The literature reviewed in this narrative review was identified through a PubMed search conducted between June and July 2026. The search was performed using combinations of the keywords “stroke”, “virtual reality”, “upper limb”, and “rehabilitation”. English-language articles were considered, with emphasis on randomized controlled trials, systematic reviews, meta-analyses, and other clinically relevant studies. Representative basic science studies on stroke rehabilitation were also included when relevant to explain the theoretical foundations and mechanisms of VR-based rehabilitation. Articles were selected based on their relevance to the scope of this review, with particular emphasis on the clinical characteristics, practical applications, and current evidence regarding immersive and non-immersive VR for post-stroke upper limb rehabilitation.

## 2. History of Virtual Reality Training

VR has a long history. Early devices developed in the 1830s and 1840s achieved stereoscopic vision by presenting slightly different images to each eye through two separate viewing windows [4]. In the 1960s, the first head-mounted display (HMD), which formed the basis of modern VR technology, was developed, and VR applications subsequently expanded to flight simulation and immersive cinema during the latter half of the twentieth century [5].

The medical application of VR began to emerge in the mid-1990s, and the number of related studies has increased substantially over the past three decades. VR has been applied in various medical fields, including surgical simulation for surgeons and medical students, the treatment of psychiatric disorders such as post-traumatic stress disorder and social anxiety disorder, and the management of chronic diseases [6,7,8,9]. In rehabilitation medicine, VR has been used to treat physical impairments, such as upper limb hemiparesis and gait disorders, as well as cognitive impairments, including cognitive dysfunction and unilateral spatial neglect, after neurological injury [10,11,12,13].

## 3. Classification of Virtual Reality Training

**Figure 1:**
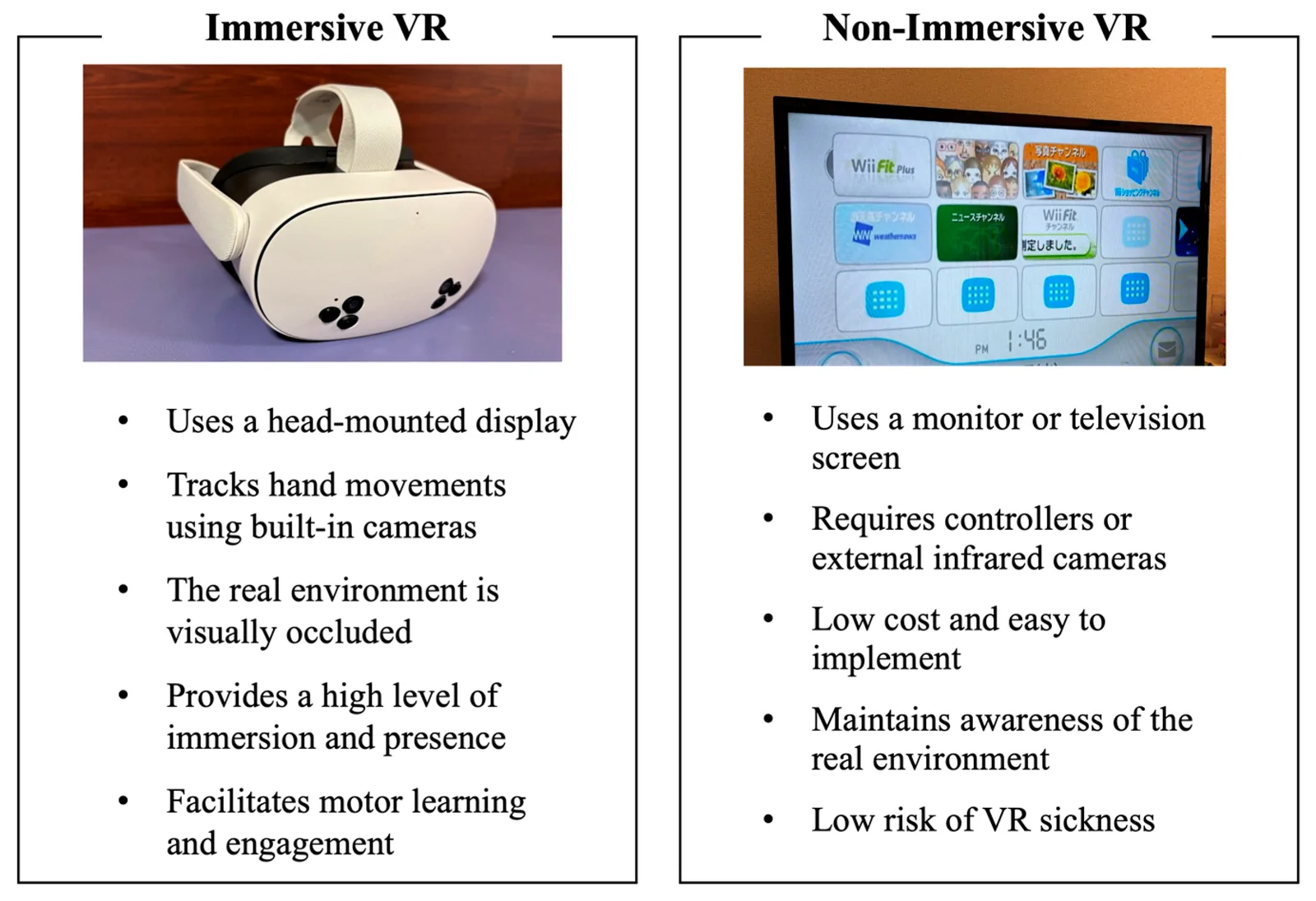
Characteristics of immersive and non-immersive virtual reality (VR). Examples of an immersive VR system (Meta Quest 3S; Meta Platforms, Menlo Park, CA, USA) and a non-immersive VR system (Wii; Nintendo, Kyoto, Japan) are shown.

VR is generally classified into two types (Figure 1): immersive VR and non-immersive VR. Many immersive VR systems used in post-stroke rehabilitation employ an HMD, which blocks external visual information and provides users with a highly immersive virtual environment. In addition, immersive VR allows real-time interaction based on head and body movements and provides a first-person perspective, thereby enhancing the user’s sense of presence. These characteristics enable users to maintain greater attention to the virtual environment and facilitate active engagement during training.

In contrast, non-immersive VR enables users to interact with virtual environments through a monitor or television screen while maintaining visual awareness of the real world. Although the level of immersion is lower than that of immersive VR, non-immersive VR is generally easier to implement, associated with a lower risk of VR sickness, and more suitable for home-based rehabilitation and telerehabilitation.

## 4. Theoretical Foundations of Virtual Reality Training

**Figure 2:**
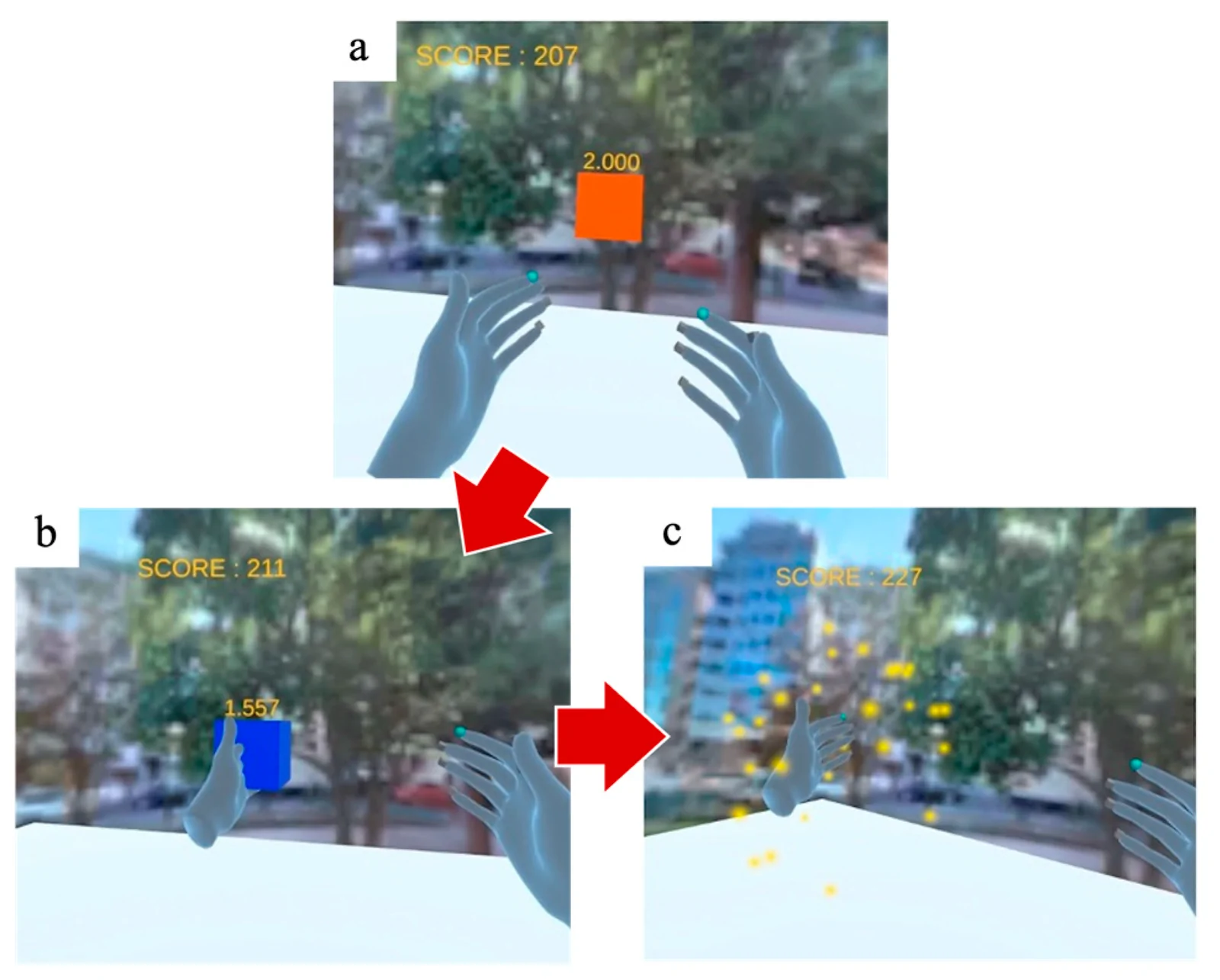
Example of visual feedback used to facilitate motor learning in virtual reality. The participant’s hands and orange target cubes are projected in the virtual environment (**a**). When the participant successfully reaches and touches a target, the target changes from orange to blue to indicate successful contact (**b**). If contact is maintained for 2 s, the target disappears with a small yellow explosion accompanied by a reward sound (**c**). The"SCORE" displayed at the top of the screen represents the cumulative duration of successful target contact.

The theoretical foundations of VR-based rehabilitation include motor learning, neuroplasticity, and enhanced motivation. Another important characteristic of VR is its ability to provide multisensory feedback. In addition to visual and auditory feedback, some VR systems are capable of providing haptic feedback using wearable devices, such as haptic gloves. Such feedback may further enhance sensorimotor integration by providing tactile information during task performance. Although haptic feedback represents an important component of some VR systems, it has been incorporated into only a limited number of rehabilitation systems for post-stroke upper limb hemiparesis. Therefore, the present review primarily focuses on visual and auditory feedback, which are the most commonly implemented modalities in current clinical practice. In particular, visual and auditory feedback play an important role in helping patients immediately recognize the outcome of their movements and correct movement errors. For example, VR systems can provide real-time visual feedback on movement success, hand position, movement trajectory, and task performance (Figure 2). In addition, auditory cues, such as sound effects or reward sounds presented after successful task completion, can further enhance patients’ attention and motivation. Such immediate and intuitive feedback facilitates error correction during motor learning and may contribute to more efficient acquisition of motor skills. Basic research has suggested that high-frequency and sustained training are essential for motor learning and neural reorganization after stroke. Bell et al. compared functional recovery among three groups of mice with experimental cerebral infarction: a high-frequency training group that received task-specific training twice daily, a low-frequency training group that trained once daily, and a non-training control group, with rehabilitation initiated 5 days after stroke onset [14]. The high-frequency training group showed significantly earlier improvement in motor function than the other groups [14]. These findings suggest that repetitive training at a higher frequency may strengthen neuronal connections and promote cortical reorganization in brain regions associated with the paretic limb. However, these functional gains were not maintained after training was discontinued, and motor function gradually declined in all groups over time. Although findings from animal models cannot be directly translated into specific clinical dosing recommendations, these results suggest that stroke rehabilitation should incorporate not only frequent repetitive training but also long-term continuation of training to maintain functional recovery. Most VR-based rehabilitation programs incorporate elements of gamification. Patients can actively participate in training while enjoying the tasks, and quantitative feedback on their performance enables them to recognize their own progress. Consequently, improvements in intrinsic motivation and self-efficacy may encourage continued participation in rehabilitation. By combining engaging task design with immediate multisensory feedback, VR helps maintain patients’ motivation and adherence throughout the rehabilitation process.

Among the different types of VR, immersive VR is characterized by a high level of immersion and an enhanced sense of presence within a virtual environment [15]. By limiting visual input from the real world using an HMD, immersive VR enables patients to maintain greater attention to the training task. In addition, first-person virtual experiences enhance the sense of agency and body ownership, thereby promoting active engagement in motor performance. These experiences also contribute to developing a sense of presence, as if individuals were physically present within the virtual environment. The degree of this sense of presence has been reported to be associated with increased frontal midline theta (FM-θ) activity [16]. FM-θ is associated with sustained attention and cognitive engagement during task performance, and its enhancement is considered one of the neural mechanisms underlying motor learning [16].

In contrast, non-immersive VR allows patients to interact with virtual environments while maintaining visual awareness of the real-world environment. Although it provides a lower level of immersion than immersive VR, it is easier to implement in both clinical and home-based settings and generally requires less expensive equipment.

Although immersive VR offers several advantages, VR sickness remains an important consideration when using HMD. VR sickness is primarily caused by a mismatch between visual and vestibular sensory inputs and may result in symptoms such as dizziness, blurred vision, and difficulty focusing [17]. Previous studies have reported that the risk of VR sickness increases when HMDs are used continuously for more than 10 min [17], and symptom severity tends to increase with longer exposure times [18]. However, both the incidence and severity of VR sickness are strongly influenced by device performance and application design, highlighting the importance of careful patient assessment. Most symptoms are mild and transient and resolve after a short period of rest, although they may occasionally interfere with the continuation of training. In contrast, because non-immersive VR does not block visual input from the real environment, the risk of VR sickness is considerably lower.

## 5. Clinical Applications of Virtual Reality Training for Post-Stroke Upper Limb Hemiparesis

### 5.1. Immersive Virtual Reality

With recent advances in HMD technology, the clinical application of immersive VR for post-stroke upper limb hemiparesis has expanded rapidly. Mekbib et al. evaluated an immersive VR program combined with mirror therapy in patients with moderate-to-severe upper limb hemiparesis after stroke [19]. The combined intervention, when provided in addition to conventional occupational therapy, resulted in significantly greater improvements in upper limb function than conventional therapy alone [19]. Notably, participants performed an average of 371 grasping movements during each 1-h training session, suggesting that a large amount of voluntary repetitive training may have contributed to functional recovery.

**Figure 3:**
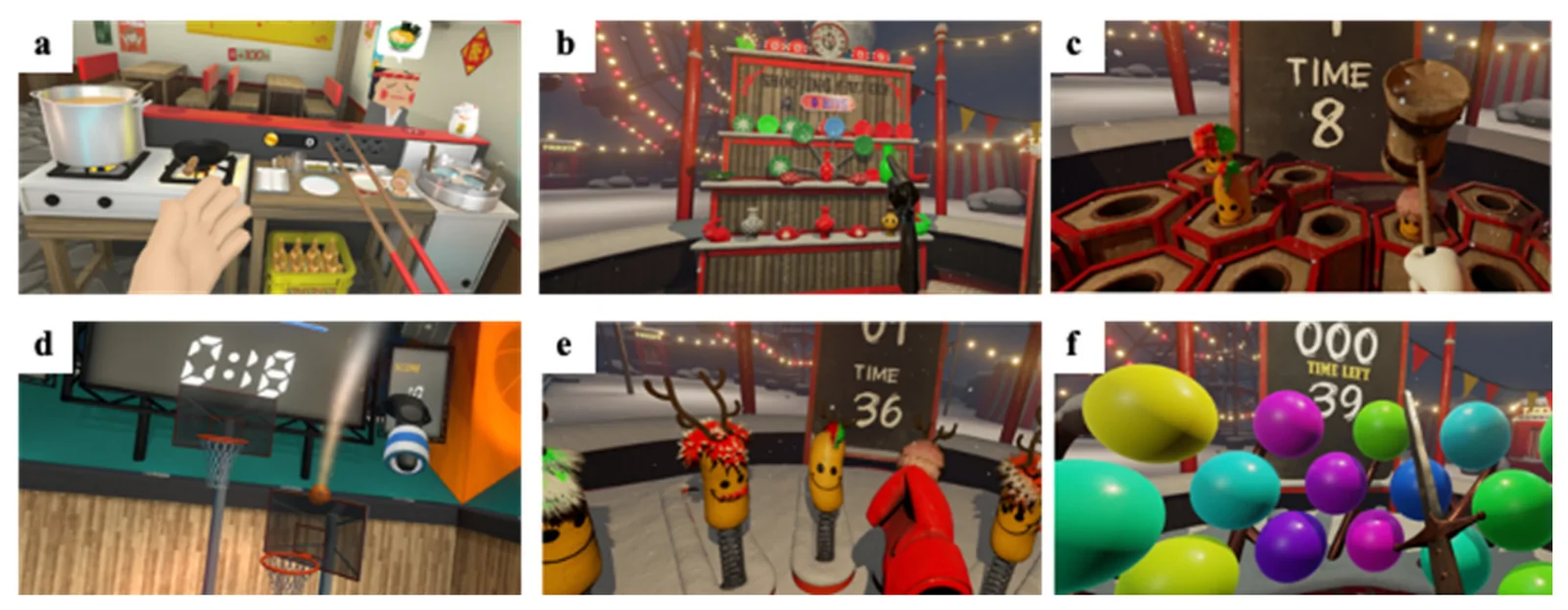
Examples of immersive virtual reality–based upper limb training tasks. (**a**) Cooking task; (**b**) shooting task; (**c**) whack-a-mole task; (**d**) basketball task; (**e**) boxing task; and (**f**) balloon-popping task. Reproduced from Huang et al. [20] under the terms of the Creative Commons Attribution 4.0 International License (CC BY 4.0).

Another important advantage of immersive VR is its ability to reproduce ADL-related tasks within a virtual environment. Huang et al. developed an immersive VR training program that simulated tasks such as cooking in a virtual kitchen and playing basketball, performed using either the paretic upper limb alone or both upper limbs (Figure 3) [20]. In a randomized controlled trial (RCT) involving patients with subacute stroke, the immersive VR group showed significantly greater improvements in upper limb function and the Barthel Index than the group receiving conventional therapy [20]. These findings suggest that motor skills acquired through immersive VR training may transfer to real ADL. Peralta-Wieland et al. reported the feasibility of an immersive VR system specifically designed for upper limb rehabilitation in patients with acute and subacute stroke [21]. The system enables repetitive training of ADL-related upper limb tasks while allowing the difficulty level to be adjusted according to each patient’s functional ability. For example, therapists can modify the number and type of reaching targets, select static or moving targets, and adjust the reaching direction to provide individualized task-oriented training. Although the efficacy of this approach has not yet been established because of the small sample size, the intervention demonstrated excellent feasibility, with an adherence rate of 98% and a User Satisfaction Evaluation Questionnaire (USEQ) score of 81.6 [21]. These promising feasibility results warrant further investigation of its clinical effectiveness in larger RCTs. George et al. conducted an RCT comparing immersive VR with non-immersive VR for upper limb rehabilitation in individuals with subacute post-stroke hemiparesis [22]. Both interventions targeted similar shoulder- and upper limb-oriented task-specific movements in combination with conventional therapy. Participants in both groups underwent 45-min VR training sessions three times per week for 6 weeks. Although both groups improved, immersive VR produced significantly greater improvements in upper limb function (Fugl–Meyer Assessment [FMA] and Wolf Motor Function Test) and stroke-specific QOL, with superior retention of these benefits at the 6-month follow-up [22].

Huang et al. further investigated the neural mechanisms underlying these functional improvements using resting-state functional magnetic resonance imaging (fMRI). Following immersive VR training, functional connectivity increased in the ipsilesional premotor cortex and primary motor cortex, together with enhanced connectivity between the visual cortex and the superior parietal lobule [23]. These findings suggest that immersive VR may facilitate reorganization of the sensorimotor network and promote functional recovery through neuroplasticity.

More recently, immersive VR has also been integrated with other rehabilitation approaches. Errante et al. developed an immersive VR training program incorporating action observation therapy (AOT) [24]. In this program, patients first observed upper limb movements performed by another person and then immediately trained the same tasks within the virtual environment, allowing a smooth transition from action observation to motor execution. In their RCT, the group receiving immersive VR combined with AOT demonstrated significantly greater improvements in the Box and Block Test than the immersive VR-only group, and these benefits were maintained at the 6-month follow-up [24]. The high level of immersion provided by immersive VR may facilitate the transition from action observation to motor imagery, thereby enhancing the effectiveness of subsequent repetitive training.

Despite these advantages, several limitations of immersive VR remain. A recent meta-analysis by Soleimani et al. suggested that immersive VR is particularly effective for improving gross motor function, whereas non-immersive VR may be more suitable for fine motor training [25]. One possible explanation is the limited accuracy of current immersive VR systems in detecting subtle finger movements. Further advances in hand-tracking and haptic feedback technologies are expected to enable more precise assessment and training of upper limb movements.

Overall, compared with non-immersive VR, immersive VR offers a higher level of presence and may provide greater improvements in upper limb function and ADL by facilitating task-oriented training in highly engaging virtual environments. However, immersive VR generally requires HMDs and dedicated equipment, which may increase implementation costs and limit accessibility in some clinical settings. Accordingly, immersive and non-immersive VR should be regarded as complementary rehabilitation approaches rather than competing technologies, with the choice depending on rehabilitation goals, available resources, and patient characteristics.

**Figure 4:**
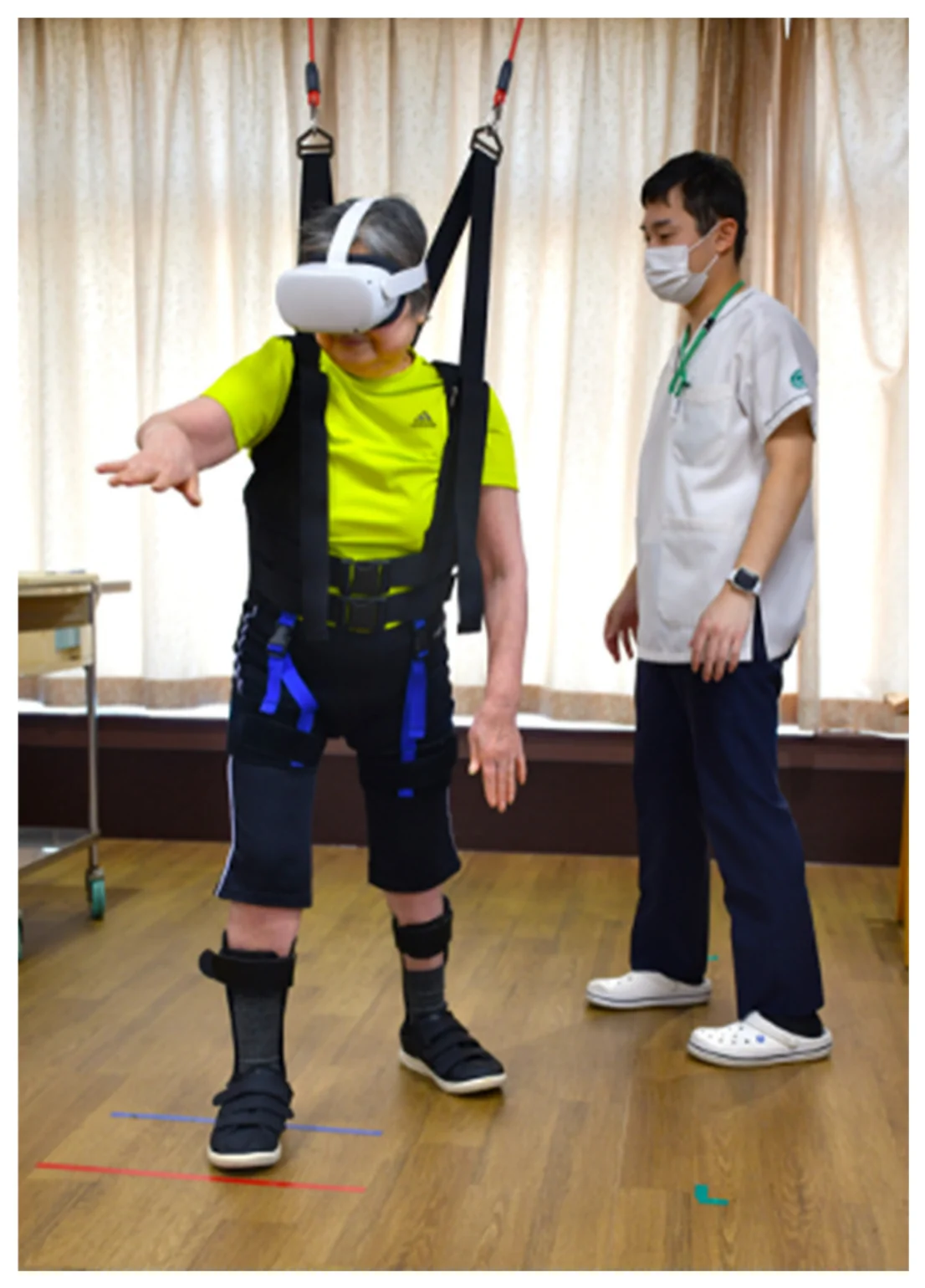
Standing balance training using immersive virtual reality (VR) combined with a body weight-supporting system. By combining immersive VR with a body weight-supporting system, patients can safely perform task-oriented standing balance training with a higher level of challenge and repetition.

At our institution, we have developed an immersive VR training program combined with a body weight-supporting system for standing balance training and are currently applying it primarily to patients with stroke and neurological disorders who present with gait and balance impairments (Figure 4) [26]. Future developments are expected to expand its application to upper limb training and to integrate additional technologies, such as functional electrical stimulation (FES) and robotic assistance, to establish more comprehensive rehabilitation programs.

### 5.2. Non-Immersive Virtual Reality

Early studies of non-immersive VR rehabilitation frequently used commercially available gaming systems such as the Wii (Nintendo, Kyoto, Japan) and Xbox Kinect (Microsoft, Redmond, WA, USA) for upper limb rehabilitation [27,28]. These systems are relatively inexpensive, easy to implement, and can enhance patient motivation through game-based activities. However, because they were originally developed for healthy users, they often lack task customization and difficulty adjustment tailored to the functional impairments of stroke patients. Consequently, some studies have reported that their therapeutic effects are comparable to those of conventional therapy alone [27]. Therefore, recent studies have increasingly focused on combining non-immersive VR with other rehabilitation interventions.

Lee et al. compared the effects of VR alone, transcranial direct current stimulation (tDCS) alone, and combined VR plus tDCS in patients with subacute stroke who had upper limb hemiparesis within 4 weeks after stroke onset [29]. The combined VR plus tDCS intervention produced the greatest improvement in upper limb function [29]. This superior effect may be attributable to the synergistic interaction between enhanced cortical excitability induced by anodal tDCS and the facilitation of the corticospinal pathway together with reduced interhemispheric inhibition induced by VR training. Guo et al. also reported that combining tDCS with robot-assisted upper limb training based on non-immersive VR improved both upper limb function and brain activity [30]. Furthermore, a recent meta-analysis demonstrated that the combination of non-immersive VR and tDCS is more effective for improving upper limb function than conventional therapy alone [31].

Shin et al. investigated changes in upper limb function and brain activity using the Rapael Smart Glove (Neofect, Seongnam, Republic of Korea), a wearable device that provides real-time measurement and feedback of finger movements, in combination with non-immersive VR [32]. Compared with conventional occupational therapy, the intervention resulted in significantly greater functional improvement. Functional near-infrared spectroscopy (fNIRS) analysis also demonstrated increased cerebral blood flow in the ipsilesional supplementary motor area [32]. Similar home-based training using the same device has also been reported to be effective [33]. In addition, studies have combined non-immersive VR with FES applied to the finger extensor muscles to further enhance upper limb recovery [34]. Compared with immersive VR, non-immersive VR is associated with fewer adverse effects, including VR sickness and falls. It can also be readily combined with other rehabilitation interventions. Therefore, it is considered a promising approach for home-based rehabilitation and telerehabilitation to support the long-term maintenance of upper limb function.

## 6. Clinical Evidence of Virtual Reality Training for Post-Stroke Upper Limb Hemiparesis

A recent Cochrane systematic review included 190 RCTs evaluating VR-based rehabilitation after stroke, many of which investigated non-immersive VR, including 66 studies using commercially available gaming systems [35]. Among the studies evaluating upper limb rehabilitation, a meta-analysis of 21 RCTs involving 689 participants demonstrated that adding VR to conventional therapy resulted in significantly greater improvements in upper limb function than conventional therapy alone (standardized mean difference [SMD], 0.42; 95% confidence interval [CI], 0.26–0.58) [35]. To further clarify the therapeutic effects of immersive VR, several meta-analyses have specifically focused on this approach. For example, Kiper et al. conducted a meta-analysis including 10 studies with 324 participants and reported that immersive VR resulted in significantly greater improvements in upper limb motor function than conventional therapy, with a mean difference of 6.33 points on the FMA (95% CI, 4.15–8.50) [10]. Furthermore, Hao et al. performed a network meta-analysis including 20 RCTs involving 813 participants and compared immersive VR, rehabilitation-specific non-immersive VR, commercial gaming systems, and conventional therapy. Immersive VR was ranked as the most effective intervention for improving upper limb function, showing the largest effect size compared with conventional therapy (SMD, 1.38; 95% CI, 0.55–2.20). Rehabilitation-specific non-immersive VR also demonstrated significantly greater improvements in upper limb function than conventional therapy, although the effect size was smaller (SMD, 0.84; 95% CI, 0.12–1.57) [36].

More recent meta-analyses including 30 RCTs involving 1661 participants have further suggested that immersive VR is more effective than non-immersive VR in improving ADL after stroke. Specifically, immersive VR demonstrated a larger effect on ADL (SMD, 0.54; 95% CI, 0.13–0.95) than non-immersive VR (SMD, 0.17; 95% CI, 0.02–0.36) [37].

In addition to studies evaluating the clinical effectiveness of immersive VR, recent research has increasingly focused on its usability, feasibility, and adverse effects. Lygouras et al. conducted a scoping review of 20 studies evaluating the usability and feasibility of immersive VR for post-stroke upper limb training using measures such as the System Usability Scale (SUS), USEQ, and Simulator Sickness Questionnaire (SSQ) [38]. Across the included studies, both patients and therapists generally reported high levels of satisfaction and good usability, whereas adverse effects were limited to mild symptoms of VR sickness. These findings support the clinical feasibility and usability of immersive VR for post-stroke upper limb rehabilitation.

Taken together, these findings consistently support the effectiveness of immersive VR for improving upper limb function and ADL after stroke and suggest that it may provide greater therapeutic benefits than conventional therapy and non-immersive VR in selected clinical settings. Nevertheless, the available evidence remains heterogeneous with respect to intervention protocols, participant characteristics, and outcome measures, highlighting the need for further standardized multicenter studies.

## 7. Recommended Therapeutic Protocol of Virtual Reality Training

To maximize the benefits of VR training, appropriate training intensity and task design are essential. Xu et al. investigated factors influencing the effectiveness of VR training for improving upper limb function after stroke, including age, training duration, and training frequency. Their findings suggested that training at least four times per week, with sessions lasting approximately 1 h for a minimum of 4 weeks, was effective for improving upper limb function. Furthermore, training sessions of approximately 2 h were recommended when the primary goal was to improve ADL [39]. These findings support the importance of high-frequency, repetitive practice for promoting motor learning after stroke.

In addition to optimizing the training protocol, appropriate monitoring of VR-related adverse events is important for the safe implementation of immersive VR. The SSQ is recommended for assessing VR-related adverse events [40,41]. In clinical practice, regular assessment using the SSQ may help clinicians adjust training duration and schedule appropriate rest periods according to each patient’s condition. In addition, some patients, particularly older adults and those with cognitive impairment, may experience anxiety or reluctance toward wearing an HMD. Therefore, clinicians should provide sufficient explanations and introduce immersive VR gradually while carefully evaluating each patient’s suitability for this training approach.

## 8. Future Directions of Virtual Reality Training for Post-Stroke Hemiparetic Patients

VR-based rehabilitation for patients with post-stroke upper limb hemiparesis has developed rapidly in recent years and has attracted considerable attention as a novel rehabilitation approach. However, several challenges remain for the broader implementation and further development of VR-based training. First, most clinical studies have been conducted in Europe and China, and substantial heterogeneity exists in study design, intervention protocols, and patient characteristics. Therefore, large-scale, multicenter RCTs are needed to better establish the clinical efficacy of VR training and identify the patient populations most likely to benefit from this approach. Second, intervention protocols should be further standardized with respect to training duration, training frequency, task design, and outcome measures to improve comparability across studies and facilitate the accumulation of high-quality evidence. Third, reducing equipment costs, providing appropriate training for rehabilitation professionals, and establishing reimbursement systems that support the routine use of VR-based rehabilitation will be important for its broader implementation in clinical practice. In Japan, addressing these issues will be essential for promoting the widespread adoption of VR-based rehabilitation.

Future research should also focus on strategies to further enhance the therapeutic effects of VR-based rehabilitation. Combining VR with other rehabilitation technologies, such as FES, robotic assistance, and devices that provide cutaneous sensory feedback, may further facilitate motor learning and neuroplasticity. In addition, expanding the use of home-based VR training may increase training frequency and overall intervention duration, thereby enabling more intensive and sustained rehabilitation. Further studies are warranted to determine whether these approaches lead to greater long-term improvements in upper limb function after stroke.

The role of VR in stroke rehabilitation is expected to continue expanding in the future. However, VR training should be regarded as a complementary rehabilitation strategy rather than a replacement for conventional therapy. The unique features of VR, including its high level of immersion, ability to enhance motivation, and capacity to facilitate repetitive training, have the potential to promote motor learning and neuroplasticity after stroke. With continued technological advances and the accumulation of high-quality clinical evidence, VR-based rehabilitation is expected to become an increasingly important component of rehabilitation strategies for post-stroke upper limb hemiparesis.

## Data Availability

No new data were created or analyzed in this study. Data sharing is not applicable to this article.

## Abbreviations

The following abbreviations are used in this manuscript:

VR	Virtual Reality
ADL	Activities of Daily Living
QOL	Quality of Life
HMD	Head-Mounted Display
RCT	Randomized Controlled Trial
USEQ	User Satisfaction Evaluation Questionnaire
FMA	Fugl–Meyer Assessment
fMRI	functional Magnetic Resonance Imaging
AOT	Action Observation Therapy
FES	Functional Electrical Stimulation
tDCS	transcranial Direct Current Stimulation
fNIRS	functional Near-Infrared Spectroscopy
SMD	Standardized Mean Difference
CI	Confidence Interval
SUS	System Usability Scale
SSQ	Simulator Sickness Questionnaire
